# A natural small molecule, catechol, induces c-Myc degradation by directly targeting ERK2 in lung cancer

**DOI:** 10.18632/oncotarget.9223

**Published:** 2016-05-07

**Authors:** Do Young Lim, Seung Ho Shin, Mee-Hyun Lee, Margarita Malakhova, Igor Kurinov, Qiong Wu, Jinglong Xu, Yanan Jiang, Ziming Dong, Kangdong Liu, Kun Yeong Lee, Ki Beom Bae, Bu Young Choi, Yibin Deng, Ann Bode, Zigang Dong

**Affiliations:** ^1^ The Hormel Institute, University of Minnesota, MN, USA; ^2^ Program in Biomedical Informatics and Computational Biology, University of Minnesota, Minneapolis, MN, USA; ^3^ The China-US (Henan) Cancer Institute, Zhengzhou, Henan, China; ^4^ Cornell University, NE-CAT, Argonne, IL, USA; ^5^ The Pathophysiology Department, The School of Basic Medical Sciences, Zhengzhou University, Zhengzhou, Hunan, China; ^6^ The Affiliated Cancer Hospital, Zhengzhou University, Zhengzhou, Henan, China; ^7^ The Collaborative Innovation Center of Henan Province for Cancer Chemoprevention, Zhengzhou, China; ^8^ Pharmaceutical Science and Engineering, School of Convergence Bioscience and Technology, Seowon University, Cheongju, Chungbuk, South Korea

**Keywords:** catechol, lung cancer, ERK2, c-Myc, natural compound

## Abstract

Various carcinogens induce EGFR/RAS/MAPK signaling, which is critical in the development of lung cancer. In particular, constitutive activation of extracellular signal-regulated kinase 2 (ERK2) is observed in many lung cancer patients, and therefore developing compounds capable of targeting ERK2 in lung carcinogenesis could be beneficial. We examined the therapeutic effect of catechol in lung cancer treatment. Catechol suppressed anchorage-independent growth of murine KP2 and human H460 lung cancer cell lines in a dose-dependent manner. Catechol inhibited ERK2 kinase activity *in vitro*, and its direct binding to the ERK2 active site was confirmed by X-ray crystallography. Phosphorylation of c-Myc, a substrate of ERK2, was decreased in catechol-treated lung cancer cells and resulted in reduced protein stability and subsequent down-regulation of total c-Myc. Treatment with catechol induced G1 phase arrest in lung cancer cells and decreased protein expression related to G1-S progression. In addition, we showed that catechol inhibited the growth of both allograft and xenograft lung cancer tumors *in vivo*. In summary, catechol exerted inhibitory effects on the ERK2/c-Myc signaling axis to reduce lung cancer tumor growth *in vitro* and *in vivo*, including a preclinical patient-derived xenograft (PDX) model. These findings suggest that catechol, a natural small molecule, possesses potential as a novel therapeutic agent against lung carcinogenesis in future clinical approaches.

## INTRODUCTION

Lung cancer is a devastating disease with very low 5-year survival rate (17%) compared to other major types of cancers, including breast (89%), prostate (99%) and colorectal (65%). It has been the leading cause of cancer death in men and women since the 1950s and 1980s, respectively [1]. Epidemiological studies showed that many risk factors, such as smoking, alcohol and asbestos, are closely related to lung carcinogenesis. Therefore, inhibiting the signaling pathways activated by these risk factors is critical. A substantial body of information showed that ERK is overexpressed and hyperactivated in lung cancers [2].

ERK is a signaling molecule commonly activated by risk factors of lung cancer including smoking [3], alcohol [4], and asbestos [5]. ERK receives signals from many oncoproteins, including EGFR, Ras and MEK, and transmits the signals to its substrates that include c-Myc and ELK1 [6, 7]. c-Myc is a transcription factor and well-known oncogene that promotes cancer cell proliferation. ERK directly binds and phosphorylates c-Myc at Ser62 resulting in c-Myc activation and enhanced protein stability [6, 8].

The Ras-MEK-ERK signaling pathway has been reported to be important in various kinds of cancers including lung, breast, oral and colon cancer [9–12]. In addition, inhibitors that have anti-cancer effects through the Ras-MEK-ERK axis have been reported in colon, breast, pancreatic, and ovarian cancers [13–16]. We previously reported that ERK inhibition with norathyriol or caffeic acid reduced the development of solar UV- induced skin carcinogenesis [17, 18]. ERK levels are overexpressed in various lung cancer cell lines [19] and targeting ERK is essential for lung cancer prevention and therapy. Therefore, we set a goal to identify natural compounds that might directly inhibit ERK activity in lung cancer.

Catechol, also known as pyrocatechol, is a naturally-occurring compound found in fruits and vegetables such as onions, apples and olive oil [20, 21]. Some phytochemicals containing the catechol moiety, such as quercetin [22, 23], luteolin [24–26], fisetin [27, 28], procyanidin B2 [29] and 7, 3′, 4′-trihydroxyisoflavone [30], showed anti-cancer activity by directly binding with signaling molecules important in carcinogenesis. However, no report has yet elucidated the anti-carcinogenic effects or direct molecular targets of catechol.

Herein, we report that catechol directly binds to ERK2 *in vitro* and *ex vivo* and suppresses its activity. Inhibition of ERK2 by catechol resulted in decreased downstream signaling, especially the phosphorylation and stability of the oncogene *c-Myc* in murine KP2 and human H460 lung cancer cell lines. Consistent with the *in vitro* results, catechol treatment inhibited c-Myc phosphorylation in mice and significantly reduced tumor growth *in vivo*. The growth of patient-derived lung tumor xenografts was also suppressed by catechol. In this report, we demonstrated that catechol inhibits the ERKs/c-Myc signaling pathway in lung cancer thereby exhibiting anti-cancer properties.

## RESULTS

### Catechol inhibits growth of lung cancer cells without toxicity

**Figure 1:**
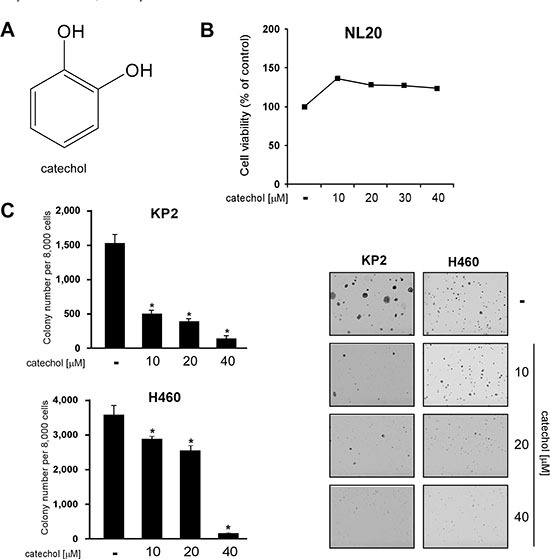
Catechol inhibits growth of lung cancer cell lines without toxicity to normal lung cells (**A**) Chemical structure of catechol. (**B**) Catechol has no effect on the viability of NL20 normal lung cells determined by MTS assay as described in Materials and Methods. (**C**) Catechol inhibits anchorage-independent growth of KP2 and H460 lung cancer cells. Photos were taken under a microscope and representative images are shown. Data are shown as mean values ± S.D. obtained from 3 independent experiments. The asterisk (*) indicates a significant difference (*p* < 0.05) between vehicle-treated and catechol-treated cells.

We determined whether catechol (Figure 1A) could inhibit cancer cell line growth without toxicity. The results of the MTS assay showed that catechol was not toxic to the normal NL20 lung cell line (Figure 1B), but inhibited the anchorage-independent growth of various types of lung cancer cells, including murine KP2 and human H460 cells (Figure 1C, Supplementary Figure 1). These results suggested that catechol preferentially kills tumor cells, and led us to further investigate the molecular mechanisms of the compound.

### ERK2 is a direct target of catechol

**Figure 2:**
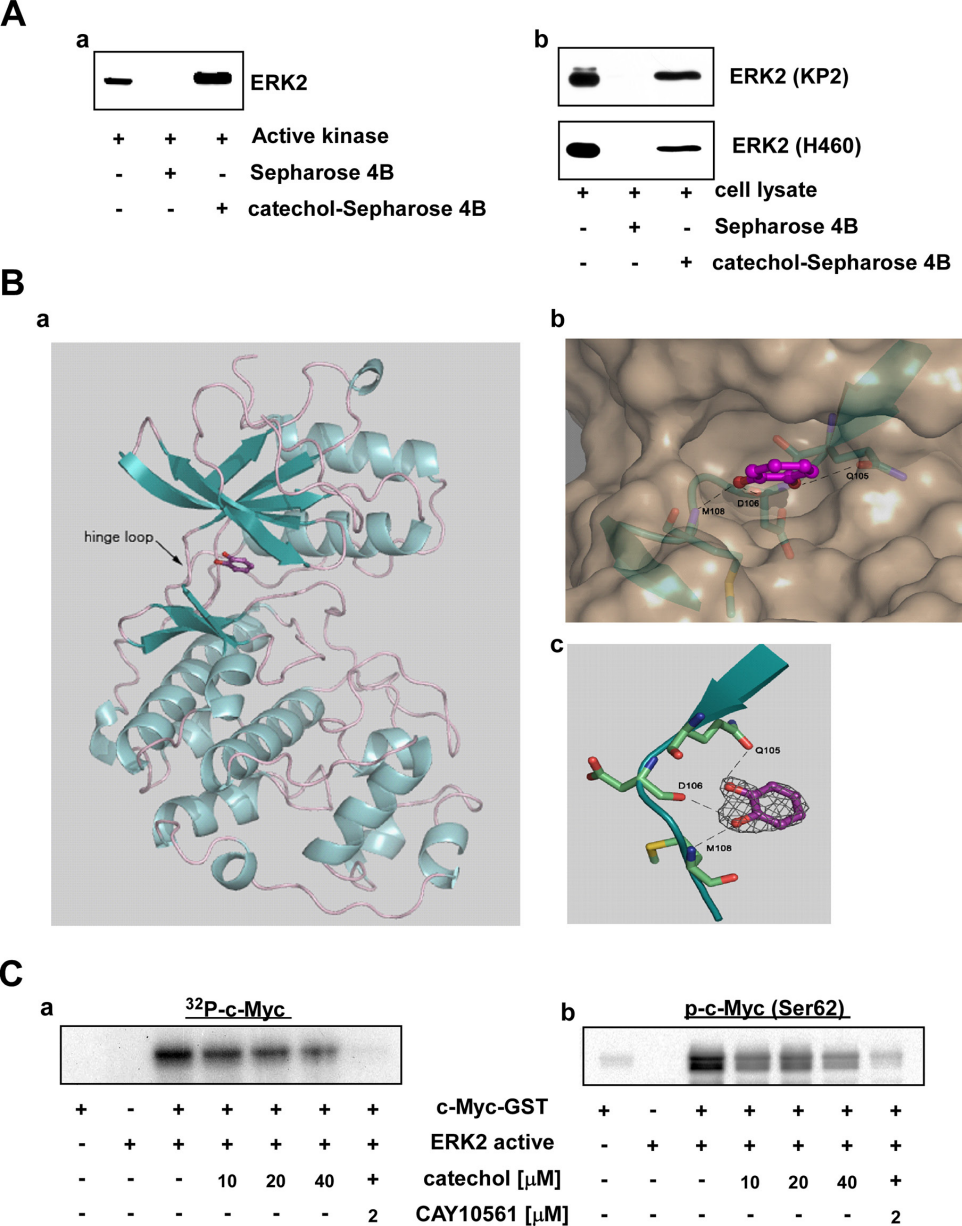
ERK2 is a direct target of catechol (**A**) Catechol directly binds with ERK2 (a) *in vitro* and (b) *ex vivo*. *In vitro* and *ex vivo* pull-down assays were performed using active kinases and KP2 or H460 cell lysates with control or catechol-conjugated Sepharose 4B beads, respectively, as described in Materials and Methods. (**B**) Catechol molecule is shown in sticks and balls in magenta with red-colored oxygen. (a) ERK2 structure in ribbon representation shows catechol bound in the ATP-binding site. (b) A Close-up view of the ERK2 active site in surface representation. The hinge loop region (residues 103–111) is shown in blue with depicted residues and Q105, D106 and M108 are shown in sticks presentation colored by elements. Catechol is located in the deep cleft. The hydrogen bonds are shown as dotted lines. The view is rotated about 90 degrees from the view on Figure, 2B–a. (c) Catechol electron density and a close-up view of binding to the hinge loop. The catechol molecule forms hydrogen bonds (dashed lines) with amino acid residues and Q105, D106 and M108 are located at the hinge loop of the ATP-binding site (residues 103–111 are shown). The residues are shown in stick presentation colored by elements. The 2|F_o_|–|F_c_| electron density map was contoured at 1.5 σ. (**C**) Catechol inhibits ERK2 kinase activity in a dose-dependent manner. *In vitro* (a) ^32^P-labeled or (b) isotope-unlabeled ERK2 kinase activity assays were performed as described in Materials and Methods.

Previously we determined the crystal structures of ERK2 complexed with caffeic acid or norathyriol [17, 18]. Because the catechol moiety is a part of these two molecules, we hypothesized that catechol could have binding affinity with ERK2. First, we performed *in vitro* and *ex vivo* pull-down binding assays, which showed that catechol directly binds with ERK2 *in vitro* (Figure 2A-a) and *ex vivo* (Figure 2A-b). Next, we determined the crystal structure of ERK2 with catechol at 2.0 Å resolution (PDB ID 4ZXT). The details of data collection and structure refinement for the ERK2/catechol complex structure are presented in Supplementary Table 1. The catechol molecule was anchored to the hinge loop of the ATP-binding site of ERK2, similar to caffeic acid in the ERK2/caffeic acid structure (PDB ID 4N0S) (Figure 2B-a). The hydroxyl groups of catechol were interacting with the main chain of D106, M108 and the side chain of Q105 located on the hinge loop (Figure 2Bb and -c). To analyze the specificity of ERK2 amino acids for the catechol moiety in the hinge loop, we compared the amino acid residues in the hinge region of the ATP-binding site from the available three-dimensional crystal structures of various kinases with their known inhibitors. The alignment showed that MAP kinases, such as p38 or JNK1/2, have different amino acid residues compared with ERK2 that underlie the specificity of catechol to the ERK2 residues, Q105, D106 and M108 (Supplementary Figure 2B). For instance, the residues in the hinge loop of N-terminal kinase domain RSK2 are completely different from those of ERK2 (Supplementary Figure 2B). Indeed, pull-down assay results with catechol-conjugated Sepharose 4B beads showed that catechol did not bind with p38, JNKs or RSK *ex vivo* (Supplementary Figure 2C). In addition, catechol did not affect the *in vitro* kinase activity of recombinant p38, JNK2 or RSK2 (Supplementary Figure 2D). To confirm that catechol inhibits ERK2 activity, we performed an *in vitro* kinase assay using a recombinant c-Myc-GST fusion protein, which is a well-known substrate of ERK2. The level of phosphorylated c-Myc was decreased dose-dependently by catechol treatment (Figure 2C-a). ERK2 can phosphorylate c-Myc at residue serine 62 (Ser62) [6, 31]; and we found that catechol strongly inhibited Ser62 phosphorylation of c-Myc (Figure 2C-a) *in vitro*. These results demonstrated that catechol directly inhibits ERK2 activity *in vitro*.

Due to the fact that ERK1 and ERK2 are 84% identical in sequence [32], we also performed a binding assay with ERK1 and catechol as well as an ERK1 *in vitro* kinase assay. Catechol was able to bind ERK1 and inhibited its kinase activity in a dose-dependent manner (Supplementary Figure 2A). Radtke, S. *et al*. and our unpublished data indicated that ERK2 expression levels are much higher compared to ERK1 levels in most lung cancer cell lines [19]. Therefore, we focused mainly on ERK2 in the following experiments presented in this study.

### Catechol reduces c-Myc phosphorylation by inhibiting ERK2 activity in lung cancer cells

**Figure 3:**
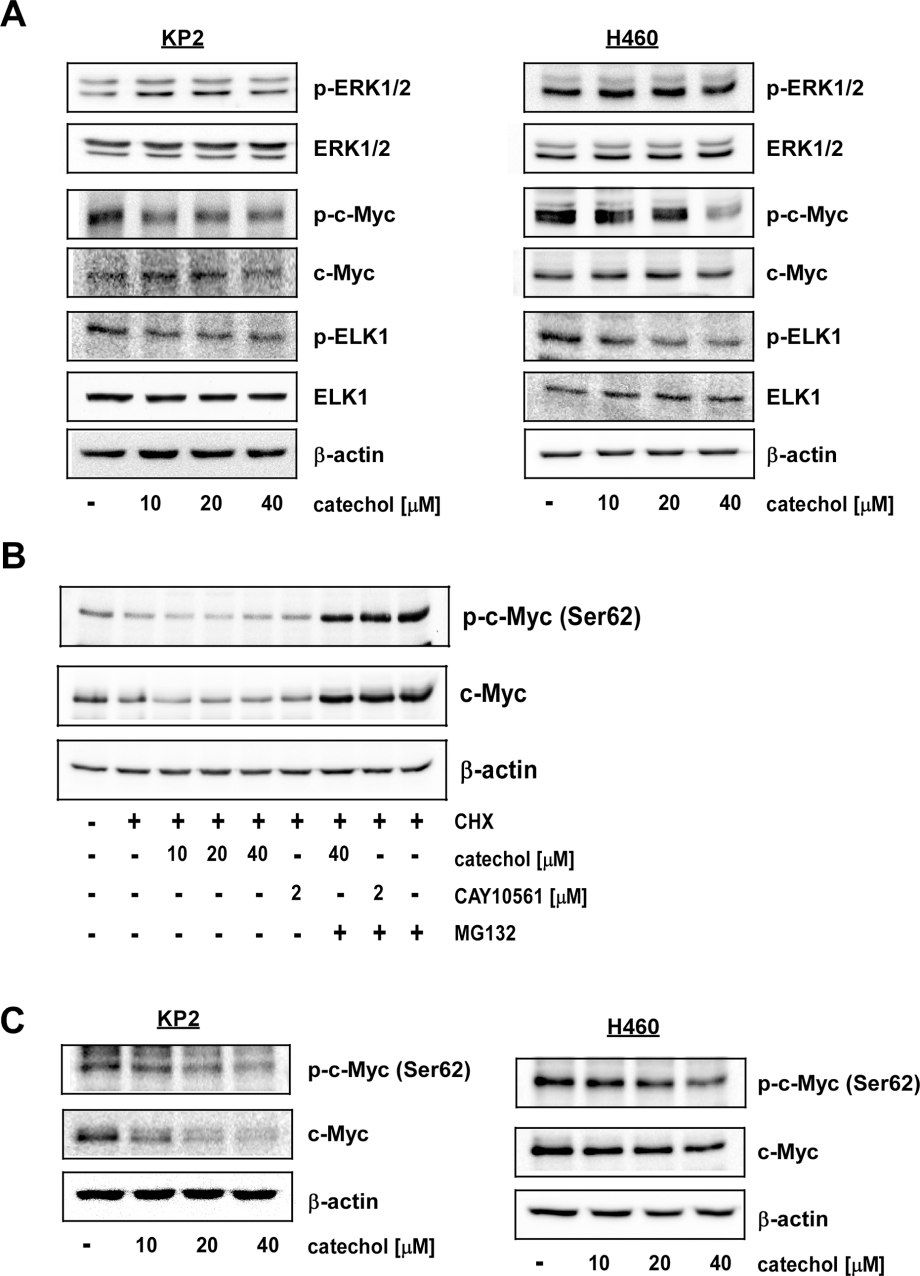
Catechol inhibits phosphorylation of c-Myc (Ser62) by targeting ERK2 Catechol inhibits phosphorylation of c-Myc (Ser62) and ELK1 (Ser383). Cells were treated with catechol (0, 10, 20, or 40 μM) for 6 h (**A** and **B**) or 24 h (**C**). Total cell lysates were subjected to Western blot analysis with specific antibodies, as indicated, to determine the levels of phosphorylated and total proteins. Representative blots from 3 independent experiments are shown. Total protein and/or β-actin are presented as loading controls.

To confirm the *in vitro* results described above, we used two cell lines, murine KP2 and human H460 lung cancer cell lines, to determine the inhibitory effect of catechol on ERK2 activity. Decreased phosphorylation of c-Myc was observed in lung cancer cells after 6 h of treatment with catechol without any changes in total c-Myc protein levels (Figure 3A). Other downstream substrates of ERKs, such as ELK1, also showed decreased phosphorylation after catechol treatment (Figure 3A). ERKs reportedly can increase c-Myc stability by phosphorylation on Ser62 [6]. Therefore, to determine the effect of catechol on c-Myc by proteasomal degradation, cells were treated with cycloheximide (CHX) in the presence or absence of MG132. CHX and MG132 are commonly used for inhibition of protein synthesis and proteasomal degradation, respectively. Because CHX blocks protein synthesis, we can show that c-Myc protein underwent proteasomal degradation after 6 h of catechol treatment due to the inhibition of ERKs activity. Results indicated that catechol induced the proteasomal degradation of c-Myc corresponding with a decreased Ser62 phosphorylation in H460 human lung cancer cells (Figure 3B). Moreover, when we treated cells with catechol and CHX in the presence of MG132, no differences were observed compared with the control. MG132 is a strong proteasome inhibitor. Therefore, even though catechol inhibited ERKs activity, which means ERKs cannot phosphorylate the c-Myc Ser62 residue, c-Myc levels were not changed by catechol treatment because proteasome activity was blocked by MG132. CAY10561, an ERKs inhibitor, also suppressed Ser62 phosphorylation and decreased c-Myc expression in H460 cells after treatment with CHX but not in the presence of MG132 (Figure 3B). Additionally, we determined that catechol might have an effect on *c-Myc* mRNA expression level at this time point. The transcript level of *c-Myc* was not changed by 6 h of catechol treatment (Supplementary Figure 3). To further confirm this result, KP2 and H460 lung cancer cells were treated with catechol for 24 h. Results indicated that c-Myc phosphorylation (Ser62) and total c-Myc protein levels were both decreased by catechol (Figure 3C). This finding suggests that catechol can disrupt the oncogenic function of c-Myc by decreasing phosphorylated c-Myc and total protein levels through inhibition of ERK2.

### Catechol induces G1 phase arrest in lung cancer cells by reducing the expression of cell cycle regulatory proteins

**Figure 4:**
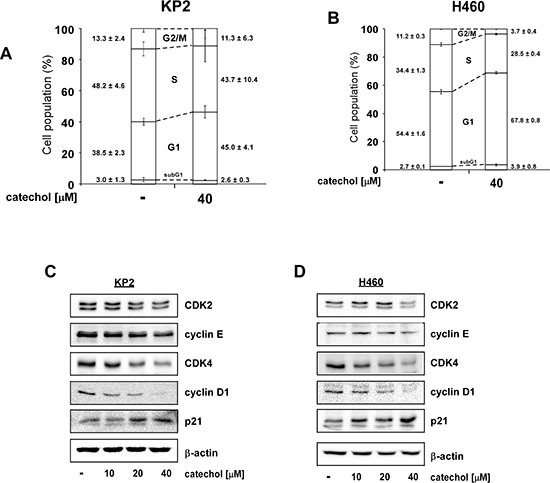
Catechol induces G1 phase arrest and reduces G1 phase-related protein expression Catechol induces G1 cell cycle arrest in (**A**) KP2 and (**B**) H460 lung cancer cells. Cells were treated with catechol (0 or 40 μM) and cell cycle analyses were performed by FACS. Data are shown as mean values ± S.D. obtained from triplicate samples from 3 independent experiments. (**C** and **D**) Catechol down-regulates G1 phase-related protein expression including CDK2, cyclin E, CDK4, cyclin D1 and up-regulates the p21 tumor suppressor protein. Cells were treated with catechol (0, 10, 20, or 40 μM) for 24 h. Cells were disrupted and subjected to Western blot analysis. Representative blots from 3 independent experiments are shown. The relative abundance of each band is normalized to β-actin.

Because c-Myc is a well-known transcription factor for cell-cycle related genes, inhibition of c-Myc levels can affect cell cycle progression [33]. Cell cycle analysis revealed that catechol induced G1-phase arrest in both KP2 (Figure 4A) and H460 (Figure 4B) lung cancer cell lines. CDK-cyclin complexes are major regulators of cell cycle and the activities of CDK-cyclin complexes are suppressed by CDK inhibitors such as the p21 and p27 proteins [34, 35]. Catechol attenuated the expression of CDK2 and CDK4 and their respective regulatory partners, cyclin E and cyclin D1 in a dose-dependent manner in KP2 (Figure 4C) and H460 (Figure 4D) lung cancer cells.

### Catechol inhibits lung tumor growth in an *in vivo* mouse model

**Figure 5:**
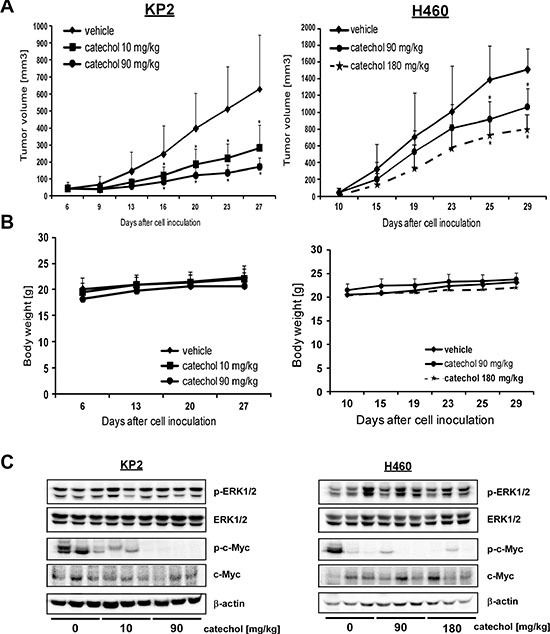
Catechol inhibits growth of xenograft tumors generated from KP2 and H460 lung cancer cells Mice were divided into 3 groups for KP2 allograft and H460 xenograft mouse models and treated as described in Materials and Methods. Groups are as follows, respectively: 1) vehicle group; 2) 10 mg/kg of catechol; and 3) 90 mg/kg of catechol for KP2; and 1) vehicle group; 2) 90 mg/kg of catechol; and 3) 180 mg/kg of catechol for H460. (**A**) Tumor volume and (**B**) body weight are shown. Data are represented as mean values ± S.D. The asterisk (*) indicates a significant difference (*p* < 0.05) between vehicle- and catechol-treated groups. (**C**) The level of phosphorylated c-Myc (Ser62) and total c-Myc expression in tumor tissues were analyzed by Western blot (*n* = 3).

To confirm that catechol has potent anti-cancer properties against tumor cells, we performed *in vivo* allograft and xenograft experiments. Athymic nude mice were injected with KP2 murine and H460 human lung cancer cells, respectively, and they were orally administered catechol. Tumor growth was analyzed for 28 days and the results showed that catechol significantly inhibited growth of KP2 allograft and H460 xenograft tumors (Figure 5A) without any change in their respective body weights (Figure 5B). Tumor tissues were collected at the end of the study and analyzed by Western blotting. The results showed that the level of phosphorylated c-Myc (Ser62) was decreased in the mouse group that had been administered catechol orally (Figure 5C). Our mouse models demonstrated that catechol has anti-carcinogenic effects against lung cancer tumor growth *in vivo*.

### Catechol suppresses lung cancer patient-derived xenograft (PDX) tumor growth

**Figure 6:**
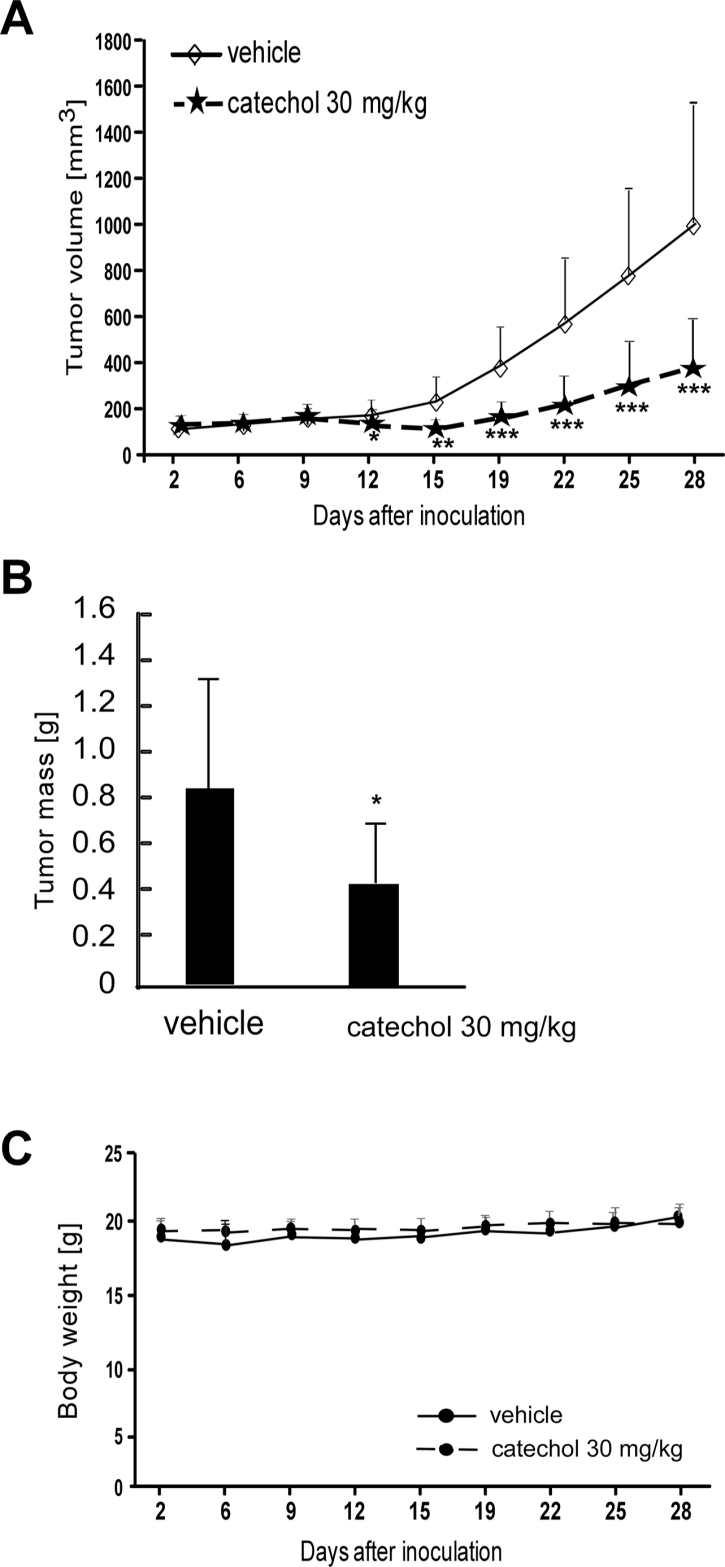
Catechol attenuates growth of lung cancer patient-derived xenograft tumors Mice were divided into 2 groups for assessing the effect of catechol on lung cancer patient-derived xenograft tumor growth. Groups are as follows: 1) vehicle group and 2) 30 mg/kg of catechol. (**A**) Tumor volume, (**B**) tumor mass and (**C**) body weights are shown. Data are represented as mean values ± S.D. Asterisks (*, **, or ***) indicate a significant difference (*p* < 0.05, 0.01, or 0.001, respectively) between vehicle-treated and catechol-treated groups.

In addition, we conducted patient-derived xenograft studies with tumors that were collected from lung cancer patients. The results showed that catechol orally administrated (30 mg/kg) to mice significantly suppressed the growth of lung cancer tumors (Figure 6A and 6B). At the same time, no changes were observed in mouse body weight (Figure 6C), indicating minimal toxicity. These data were in agreement with our previously obtained results in allograft and xenograft mice models as described above (Figure 5). The results of both in the *in vivo* lung cancer cell-tumor models and preclinical mouse PDX model demonstrate that catechol has a potential to be a clinical chemotherapeutic agent against lung cancer.

## DISCUSSION

A highly relevant and important clinical task is to properly evaluate chemicals, including carcinogens or compounds with so-called health benefits, to which humans are frequently exposed. For many years, catechol has had a reputation as a potential carcinogen based on high-dose experiments that resulted in tumor-promotion in animals [36]. Because catechol was widely used in many industrial areas, the compound was considered to be a xenobiotic, which is a foreign chemical substance found within an organism that is not normally produced by or expected to be present within that organism. Thus, establishing safety information relevant to occupational exposure to catechol was an urgent issue and, therefore, the initial risk assessment was only focused on identifying the harmful effects of the compound. However, a substantial body of information has indicated that catechol is ubiquitous in nature and has been consumed in food for decades without causing any documented adverse effects. The fact that consumers are encouraged to continue to eat fruits and vegetables that contain catechol, which has been considered to be a potential carcinogen, is paradoxical. This inconsistency led us to further evaluate catechol, which is mainly consumed in food or through smoking [37]. As further experiments were designed, we chose to study lung cancer where catechol could have either carcinogenic or therapeutic effects. Unexpectedly, we observed potent inhibitory effects of this compound against lung cancer cell lines, including KP2 and H460 cells.

Notably, tumor cells in which Kras and ERKs are highly activated were very sensitive to catechol treatment. The allograft growth of KP2 cells was more effectively inhibited by catechol treatment compared to H460 cells. KP2 cells were generated from *Kras*^LSL-G12D/wt^*/p53*^flox/flox^ mice. These mice were developed from an autochthonous mouse model of human lung cancer. *Kras*^G12D^ by itself activates a low grade of adenocarcinomas. However, the combination of *Kras*^G12D^ with the concomitant loss of both p53 alleles resulted in aggressive development of lung tumors in mice [38]. Kras is one of three members of the Ras family and is essential for normal development [39]. Kras is upstream of the Raf/mitogen-activated protein kinase (MAPKs; ERK1/2) signaling pathways and most Ras activation in tumors occurs as the result of mutations in codons 12, 13 and 61 [40] and Kras^G12D^ mutation causes continuous activation of its downstream signaling pathway. This explains why catechol was more effective *in vivo* against KP2 cells compared to H460 cells because ERKs are more highly activated in KP2 cells compared to H460 cells. This supports the idea that, clinically, catechol could be more effective in patients who express high Kras/ERKs activity.

We solved the crystal structure of the ERK2 complexed with catechol and confirmed that catechol directly binds to the ATP-binding site of ERK2. Previously, we purified recombinant non-phosphorylated ERK2 and optimized the conditions to grow protein crystals diffracting to 1.6 Å. Using these high quality ERK2 crystals, we tested several potential inhibitors and determined ERK2 structures complexed with norathyriol or caffeic acid [17, 18]. Currently, about 100 crystal structures of ERK2 have been deposited in the PDB, compared to about a dozen in 2011. Many of these structures showed that ERK2 binds to different classes of compounds. A growing interest among researchers focuses on finding ERK2 inhibitors for cancer treatment. Our current ERK2 model differs from the double phosphorylated ERK2 structure (PDB ID 2ERK) with rmsd 1.2 Å. Most of the differences are observed in the activation loop and terminus positions. At the same time, our ERK2 model is very similar to the ATP-bound ERK2 (PDB ID 4GT3) and AMP-PNP-bound ERK2 (PDB ID 4S32) models with rmsd 0.54 Å for both structures. The rmsd for the main chain atoms in the active site is in the range of 0.2–05 Å. The adenosine ring of ATP in those structures is anchored to the hinge loop by Q105 and D106 residues. Similarly, catechol interacted with the same residues and occupied the nearby adenosine ring position inside the ATP-binding site.

Recently, tumor-specific patient-derived xenograft models have emerged as a model to develop and evaluate compounds as therapeutic reagents against cancer. PDX models are biologically stable when the tumor is passaged in mice and have the advantage of maintaining specific genetic characteristics from individual cancer patients [41, 42]. Our results showed that catechol was very effective in inhibiting the growth of these tumors in mice.

To our knowledge, this is the first report of the health-beneficial effects of catechol using several models, including a patient derived xenograft model of lung cancer, and we identified a direct target of catechol to inhibit cancer growth. These results challenge the assessment of catechol as a carcinogen and provide insights of its anticarcinogenic properties. Thus, the clinical properties of catechol against cancer need to be reassessed as a health-promoting natural compound.

## MATERIALS AND METHODS

### Reagents

Catechol (1,2-dihydroxybenezene) was purchased from Sigma-Aldrich (St. Louis, MO) and active ERK2, p38, JNK2, and RSK were from Millipore (Temecula, CA). Antibodies to detect phosphorylated ERK1/2 (p-ERK1/2 Thr202/Tyr204), phosphorylated ELK1 (p-ELK1), total ELK1, total ERKs, cyclin E, p38, JNK, and RSK were purchased from Cell Signaling Technology (Beverly, MA). The antibodies against, c-Myc, CDK2, CDK4, cyclin D1, p21 and β-actin were from Santa Cruz Biotechnology (Santa Cruz, CA). The antibody against phosphorylated c-Myc (p-c-Myc Ser62) was obtained from Abcam (Cambridge, MA). CNBr-Sepharose 4B beads were from Amersham Pharmacia Biotech (Piscataway, NJ). The Cell Titer 96 Aqueous One Solution Reagent [3-(4,5-dimethylthiazol-2-yl)-5-(3-carboxymethoxyphenyl)-2-(4-sulfophenyl)-2H-tetrazolium, inner salt (MTS)] kit for the cell proliferation assay were from Promega (Madison, WI).

### Cell culture

The KP2 murine lung cancer cell line (*Kras*^LSL-G12D/wt^*;p53*^flox/flox^) was provided from Dr. Tylor Jacks (MIT, Cambridge, MA) [43]. NL20, H460, HCC827 and H1650 human lung normal and cancer cell lines were purchased from American Type Culture Collection. HCC827 GR human lung cancer cell lines were obtained from Dr. Pasi A. Jänne (Dana-Farber Cancer Institute, Boston, MA) [44]. All cells were cytogenetically tested and authenticated before freezing. All culture conditions were performed following their instructions. Gefitinib resistance of HCC827GR cells were tested before experiments. There was significant difference in the sensitivity to gefitinib compared with HCC827 cells. Cells were maintained in a 5% CO_2_, 37°C humidified incubator. Each vial of frozen cells was thawed and maintained in culture for between 10 to 20 passages.

### Cell toxicity assay

Cells were seeded (1 × 10^4^ cells/well) in 96-well plates and incubated for 24 h and then treated with different doses of catechol. After incubation for 2 days, 20 μL of Cell Titer 96 Aqueous One Solution were added and then cells were incubated for 1 h at 37°C in a 5% CO_2_ incubator. Absorbance was measured at 492 nm.

### Anchorage-independent cell transformation assay

Cells (8 × 10^3^/well) suspended in 10% Basal Medium Eagle (Sigma Aldrich, St. Louis, MO) were added to 0.3% agar with 0, 10, 20 or 40 μM catechol in a top layer over a base layer of 0.5% agar with 0, 10, 20, or 40 μM catechol. The cultures were maintained at 37°C in a 5% CO_2_ incubator for 1–2 weeks and then colonies were counted under a microscope using the Image-Pro Plus software (v.6.1) program (Media Cybernetics).

### *In vitro* and *ex vivo* pull-down assays

Recombinant ERK2 (200 ng) proteins or KP2 and H460 cell lysates (500 μg) were incubated with catechol-Sepharose 4B (or Sepharose 4B only as a control) beads (50 μL 50% slurry) in reaction buffer (50 mM Tris pH 7.5, 5 mM EDTA, 150 mM NaCl, 1 mM/L DTT, 0.01% NP- 40, and 2 mg/mL bovine serum albumin). After incubation with gentle rocking overnight at 4°C, the beads were washed 5 times with buffer (50 mM Tris, pH 7.5, 5 mM EDTA, 150 mM NaCl, 1 mM DTT, and 0.01% NP- 40) and binding was visualized by Western blotting.

### ERK2/catechol structure determination

Recombinant human ERK2 was purified and crystallized similarly as previously described in detail [17, 18]. The crystals appeared when ERK2 (6 mg/ml, in the presence of 15 mM β-mercaptoethanol) was mixed with a precipitant solution comprised of 1.2–1.4 M ammonium sulfate, 2% PEG 500 MME, and 0.1 M HEPES-NaOH pH 7.5. The ERK2 crystals were soaked in precipitant solution containing 2.5 mM catechol in 2.5% dimethyl sulfoxide (DMSO) for one week. The crystals were cryo-protected in 1.9 M ammonium sulfate, 2% PEG 500 MME, 0.1 M HEPES-NaOH pH 7.5, and 20% xylitol and then flash-cooled in liquid nitrogen. The high-resolution 2.0 Å diffraction data were collected at the Advanced Photon Source NE-CAT beamline 24ID-E using a 30 × 50 micron beam and the Quantum 315 CCD detector. X-ray diffraction data were integrated and scaled using the HKL2000 package. The structure was solved by molecular replacement using a starting model of the refined ERK2/norathyriol structure (PDB ID 3SA0) [17]. All calculations were performed using PHENIX [45]. The final refined atomic model contains ERK2 residues 7–357, no electron density was observed for the N- and C-terminus (residues 1–6 and 358–360) and a weak electron density was observed for residues 13–15 and 336–337. All figures of the structure presentations were prepared using Pymol. The coordinates and structure factors for the complex of ERK2 with catechol have been deposited in the Protein Data Bank under accession code 4ZXT.

### *In vitro* kinase assay

The kinase assay was performed according to the instructions provided by Millipore. Briefly, for kinases (ERK2, p38, JNK2 and RSK) activity analysis, the relevant active protein (100 ng) was incubated with catechol (for ERK2; 0, 10, 20 or 40 μM or for other kinases; 0 or 80 μM) for 30 min at 30°C. Then each reaction mixture was mixed with a relevantly respective substrate, isotope-unlabeled ATP and/or 10 μCi [γ-^32^P] ATP with each compound in 10 μL of reaction buffer containing 20 mM HEPES (pH 7.4), 10 mM MgCl_2_, 10 mM MnCl_2_, and 1 mM dithiothreitol (DTT). After incubation at 30°C for 30 min, the reaction was stopped by adding 4 μL protein loading buffer and the mixture was separated by sodium dodecyl sulfate-polyacrylamide gel electrophoresis (SDS-PAGE). The ^32^P-labeled c-Myc-GST product was visualized by autoradiography. Each experiment was repeated three times and the relative amounts of incorporated radioactivity were assessed by autoradiography. For the isotope-unlabeled ATP kinase assay, phospho-c-Myc (Ser62) antibodies were visualized by Western blotting.

### Western blot analysis

Cells were disrupted on ice for 40 min in cell lysis buffer (20 mM Tris pH 7.5, 150 mM NaCl, 1 mM Na_2_EDTA, 1 mM EGTA, 1% Triton X-100, 2.5 mM sodium pyrophosphate, 1 mM β-glycerophosphate, 1 mM sodium vanadate, and 1 mM PMSF [phenylmethylsulfonyl fluoride]). After centrifugation at 12,000 rpm for 10 min, the supernatant fraction was harvested as the total cellular protein extract. The protein concentration was determined using the Bio-Rad protein assay reagent (Richmond, CA). Total cellular protein extracts were separated by SDS-PAGE and transferred to polyvinylidene fluoride (PVDF) membranes in 20 mM Tris-HCl (pH 8.0), containing 150 mM glycine and 20% (v/v) methanol. Membranes were blocked with 5% non-fat dry milk in 1 x TBS containing 0.05% Tween 20 (TBS-T) and incubated with antibodies against phosphorylated (p)-ERK1/2, ERK1/2, p-c-Myc (Ser62), c-Myc, p-ELK1, ELK1, CDK2, cyclin E, CDK4, cyclin D1, p21, p38, JNKs, RSK and β-actin. Blots were washed 3 times in 1X TBS-T buffer, followed by incubation with an appropriate HRP-linked IgG. The specific proteins in the blots were visualized using the enhanced chemiluminescence (ECL) detection reagent using Image Quant LAS 4000 (GE Healthcare Bio-Science, Pittsburgh, PA).

### Cell cycle analysis

Cells were plated at a density of 3 × 10^5^ cells/dish in 60-mm dishes. After 24 h, cells were treated with 0, 10, 20, or 40 μM catechol for another 6 h. Cells were trypsinized and then stained with propidium iodide. Stained cells were subjected to fluorescence-activated cell sorting analysis using the BD FACScan (BD Biosciences, San Jose, CA).

### *In vivo* allograft and xenograft mouse models

Athymic nude mice (5 wk old) were obtained from Harlan Laboratories (Indianapolis, IN) and maintained under specific pathogen-free conditions based on the guidelines established by the University of Minnesota Institutional Animal Care and Use Committee. Mice were divided into 3 groups for KP2 allograft and H460 xenograft mouse models, respectively, as follows: 1) vehicle group (*n* = 12); 2) 10 mg/kg of catechol (*n* = 12); and 3) 90 mg/kg of catechol (*n* = 12)]; and 1) vehicle group (*n* = 6); 2) 90 mg/kg of catechol (*n* = 10); and 3) 180 mg/kg of catechol (*n* = 10)]. KP2 and H460 cells (1 × 10^6^ cells/100 μL and 2 × 10^6^ cells/100 μL, respectively) were suspended in phosphatase buffered saline (PBS) and inoculated into the right flank of each mouse. Vehicle (PBS) or catechol (in vehicle) was orally administrated each day until the study was terminated. Body weight and tumor volume were measured two times per week. Tumor volume was calculated from measurements of the individual tumor based using the following formula: tumor volume (mm^3^) = (length × width × height × 0.52). Mice were monitored until tumors reached 1–2 cm^3^ total volume, then euthanized and tumors were extracted for further analyses.

### Patient-derived xenograft (PDX) study

Seven to eight week-old female mice with severe combined immunodeficiency (SCID) (Vital River Labs, Beijing, CN) were used for this *in vivo* study. This study was approved by the Ethics Committee of Zhengzhou University (Zhengzhou, Henan, China) and the patients whose tumor samples were used in the study were completely informed and gave full consent. Mice were caged in groups of six, kept on a 12 h light/dark cycle, and given sterile food and water *ad libitum*. A tumor specimen of lung cancer tissue was obtained at the time of surgical resection from consenting patients at the 1st affiliated Hospital of Zhengzhou University. The tumor tissue was cut into 3 portions, each with a volume of 3–4 mm^3^. One portion was used for histopathological analysis, a second was used for protein analysis, and the remainder was implanted subcutaneously into the back of the neck of 3 individual SCID mice. After the tumors were expanded through at least the 3rd generation of human lung cancer tissue, they were removed and divided into pieces, and then implanted into the back of the neck of twenty-four mice for the xenograft study. Twenty-four mice were divided into 2 groups as follows: 1) vehicle control and 2) catechol at 30 mg/kg. Four days after tumor implantation, vehicle (PBS) or catechol (30 mg/kg) was administered daily by oral gavage. Tumor volume (volume = length × width^2^ × 0.5) was evaluated every 3 days by a caliper. Volume data were analyzed using SPSS 17.0 (IBM, Armonk, NY). At the end of the experiment, mice were sacrificed prior to removal of the tumors for further analyses.

### Statistical analysis

All quantitative results are expressed as mean values ± S.D. Statistically significant differences were obtained using the Student's t test or by one-way ANOVA. A *p*-value of < 0.05 was considered to be statistically significant.

## SUPPLEMENTARY MATERIALS FIGURES AND TABLES


