# Supplementary material for: A natural small molecule, catechol, induces c-Myc degradation by directly targeting ERK2 in lung cancer
Source: Oncotarget. 2016 May 7;7(23):35001–14. doi: 10.18632/oncotarget.9223 (PMC5085205; doi:10.18632/oncotarget.9223)
Supplement: Supplementary file 1 [file oncotarget-07-35001-s001.pdf]

A natural small molecule, catechol, induces c-Myc degradation by directly targeting ERK2 in lung cancer

Supplementary Materials

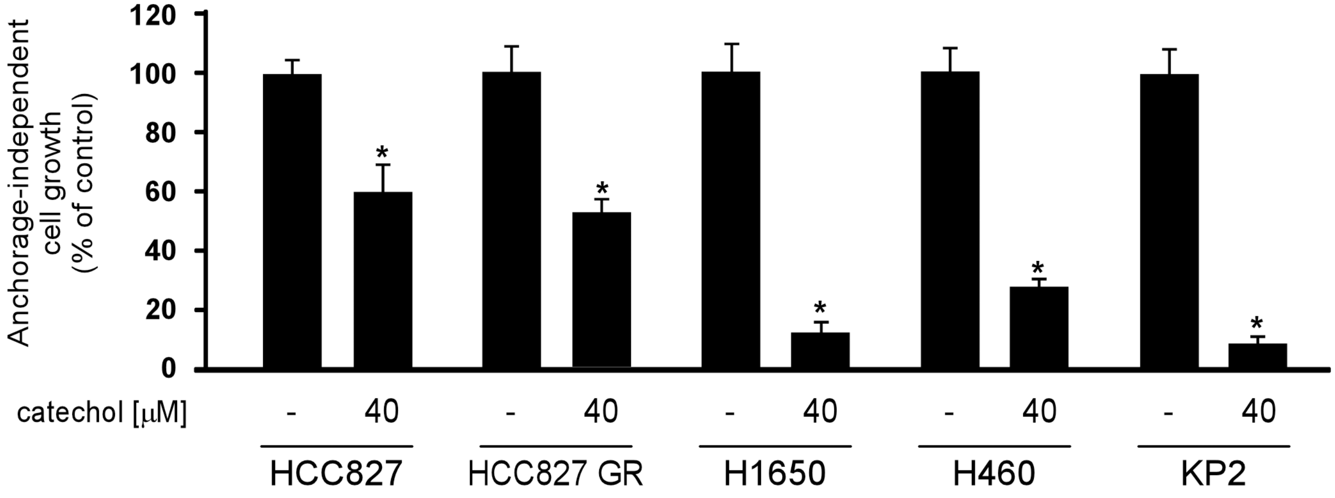

Supplementary Figure S1: Catechol inhibits growth of lung cancer cell lines. Catechol inhibits anchorage-independent growth of HCC827, HCC827GR, H1650, H460 and KP2 lung cancer cells. Data are shown as mean values ± S.D. obtained from 3 independent experiments. The asterisk (*) indicates a significant difference ($p < 0.05$) between vehicle-treated and 40 μM catechol-treated cells.

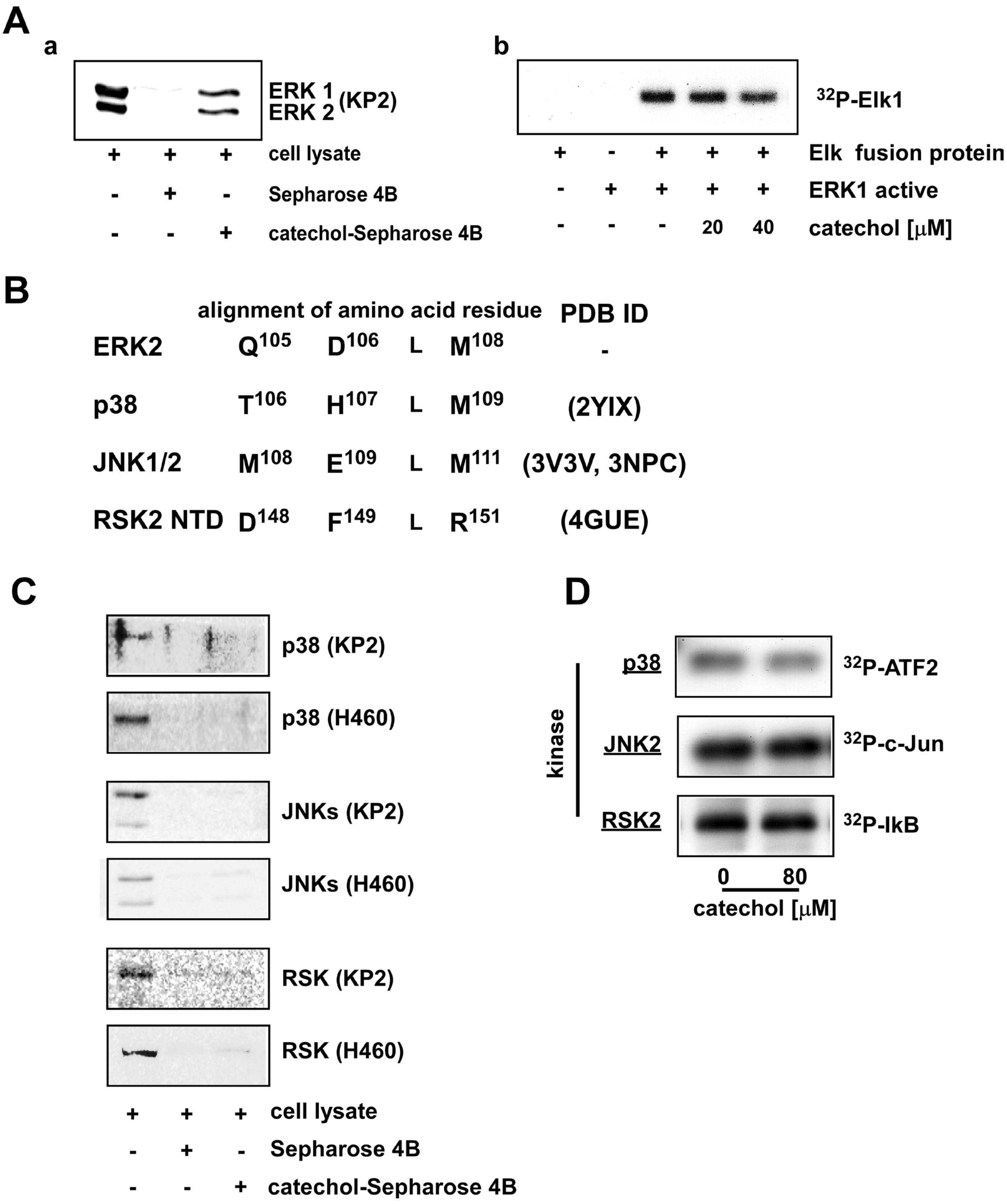

Supplementary Figure S2: ERKs are direct targets of catechol. (**A**) Catechol directly binds with ERK1 and 2 *ex vivo* (a) and inhibits ERK1 kinase activity in a dose-dependent manner (b). *Ex vivo* pull-down assays (a) and *in vitro* ^{32}P-labeled ERK1 kinase activity assay (b) were performed using active KP2 lung cancer cell lysates and ERK1 kinase, respectively, as described in Materials and Methods. (**B**) The alignment of amino acid residues in the hinge loop for ERK2 and other kinases, including p38, JNK1/2, and RSK2 NTD. (**C**) Catechol does not bind with p38, JNKs or RSK *ex vivo*. *Ex vivo* pull-down assays were performed using KP2 or H460 lung cancer cell lysates with or without catechol-conjugated Sepharose 4B beads, respectively, as described in Materials and Methods. (**D**) Catechol does not affect p38, JNK2 and RSK2 kinase activity. *In vitro* ^{32}P-labeled p38, JNK2 or RSK2 kinase assays were performed as described in Materials and Methods.

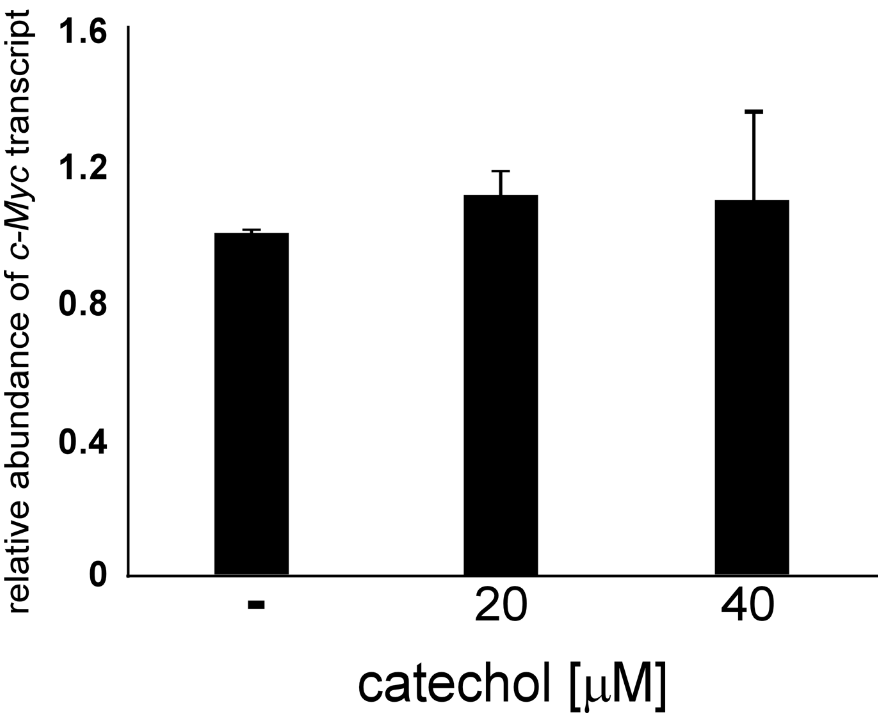

Supplementary Figure S3: Catechol does not affect *c-Myc* transcript levels at 6 h of treatment. H460 cells were treated with catechol for 6 h and then total RNA was isolated. cDNA was synthesized from the total RNA and real-time PCR was performed to determine *c-Myc* transcript levels. Each reaction was normalized by its own GAPDH (n = 3).

Supplementary Table S1: X-ray data collection and refinement statistics

**Data collection**	
Space group	$P2_1$
Cell dimensions	
a, *b*, *c* (Å)<br>β (deg.)	48.59, 69.34, 59.59<br>108.757
Resolution [a)] (Å)	44-2.0 (2.07–2.00)
R_{merge}	0.070 (0.245)
I/σ	28.9 (6.9)
Completeness (%)	99.9 (99.8)
Redundancy	4.3 (3.8)
**Refinement**	
Resolution (Å)	43.8–2.0
No. reflections	25363
$R_{work/}\ R_{free}$ [b]	0.153/0.183
No. atoms	
Protein[c]	5762
Catechol	14
Water	299
B-factors (Å^2)	
Protein	29.7
Catechol	45.9
Water	34.1
R.m.s. deviations	
Bond lengths (Å)	~0.012 Å
Bond angles (º)	~1.3 Å

[a)]Highest resolution shell is shown in parenthesis.
[b)]R_{free} was calculated from a randomly chosen 5% of reflections excluded from refinement.
[c)]including riding hydrogens.
